# LeView: automatic and interactive generation of 2D diagrams for biomacromolecule/ligand interactions

**DOI:** 10.1186/1758-2946-5-40

**Published:** 2013-08-29

**Authors:** Ségolène Caboche

**Affiliations:** 1 Protein Data Bank in Europe, EMBL-EBI, Wellcome Trust Genome Campus, Hinxton, Cambridge CB10 1SD, UK; 2 Present address: IFR142 Molecular and Cellular Medecine, INSERM, CNRS, Institut Pasteur de Lille and Univ Lille Nord de France, Lille, France

**Keywords:** Ligand, Interaction, Tool, Display, 2D diagram

## Abstract

2D diagrams are widely used in the scientific literature to represent interactions between ligands and biomacromolecules. Such schematic diagrams are very helpful to better understand the chemical interactions and biological processes in which ligands are involved. Here, a new tool for automatic and interactive generation of 2D diagrams for biomacromolecule/ligand interactions is presented. LeView (Ligand-Environment Viewer) produces customised and high-quality figures, with a good compromise between a faithful representation of the 3D data (structures and interactions) and aesthetic criteria. LeView can be freely downloaded at http://www.pegase-biosciences.com/tools/leview/.

## Background

Non-covalent interactions, such as hydrogen bonds, between ligands and biomacromolecules forming a complex provide important clues in the understanding of biological processes and the design of compounds with desirable binding properties. Schematic 2D diagrams are widely used to visualise the binding interactions of ligands, which can provide important clues about their role and function. A lot of software packages are available for visualising structure in 3D, but only a handful exists for generating 2D protein/ligand interaction diagrams. LIGPLOT [1] is the most commonly used and its successor LigPlot+ [2] includes a new interface, superposition of related diagrams and links to PyMOL and RasMol. PoseView [3] automatically generates structure diagrams of molecular complexes. A module of the commercial software MOE - Molecular Operating Environment - [4] generates schematic diagrams for protein-ligand complexes, and other commercial software vendors offer similar tools. In this paper, LeView (Ligand-Environment Viewer) is introduced. This new tool automatically detects ligands in a PDB file, generates 2D diagrams for biomacromolecule/ligand interactions and allows the user to customise diagrams through an interactive and intuitive graphical interface. LeView is written in Java and distributed as an executable file (source code is also available under GNU Public Licence, Additional file 1) in two versions: a version with an interactive graphical interface and a command-line version for use in pipelines. LeView does not require any special installation steps, can be run on all operating systems.

## Implementation

**Figure 1:**
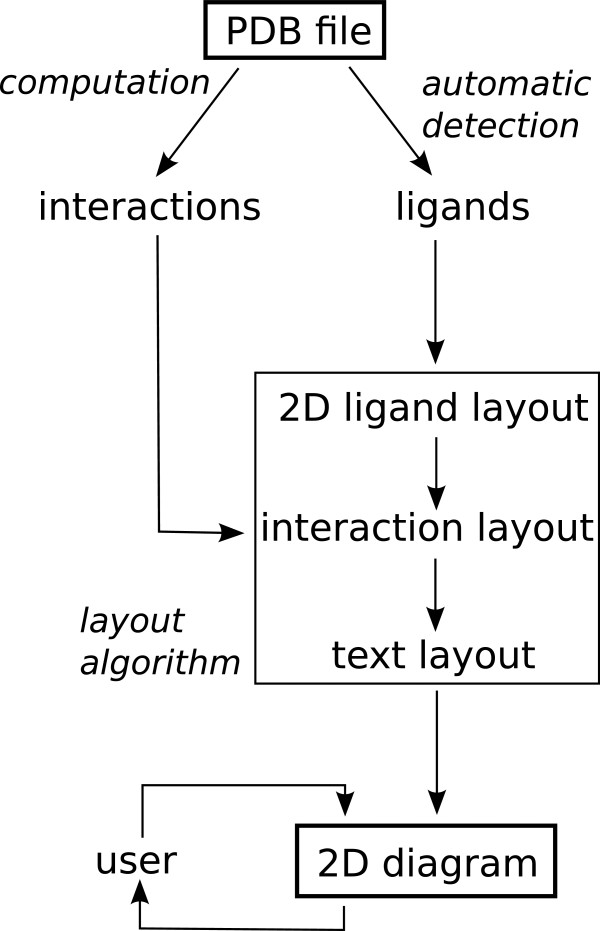
Main steps of the LeView implementation.

LeView was written in Java in order to be environment-independent, it is open-source and can be freely downloaded (GNU Public Licence). Figure 1 shows the main steps of the LeView implementation. In a first step LeView automatically identifies ligands and metal ions directly from the PDB file with an algorithm based on the method used in PDBSum [5]. Explicit residues, *i.e.* residues explicitly connected to the ligand in the PDB file, are also identified. For a ligand, the layout algorithm is then used to obtain the 2D diagram with which the user can interact via a graphical interface to customise it. The layout algorithm is divided into three steps: first, the 2D coordinates of the ligand atoms are computed, then the interactions are calculated from the PDB file and placed around the ligand and finally residue names are added to the diagram.

### Ligand layout

**Figure 2:**
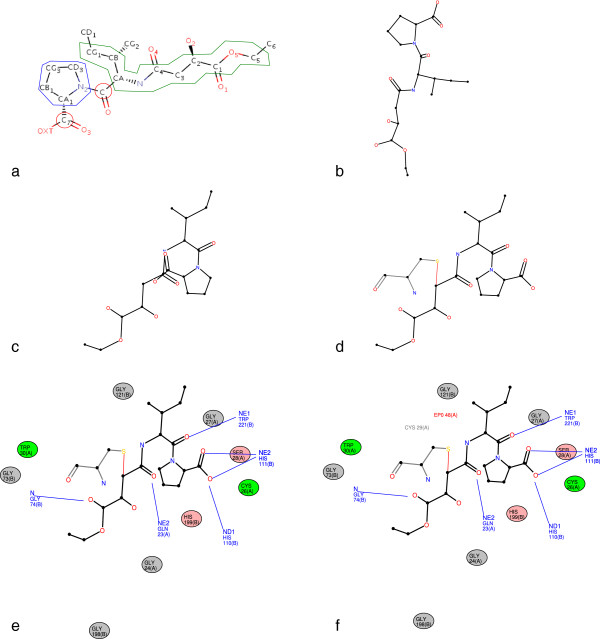
**Step-by-step diagram production.** Figure 2 shows the diagram production for epoxy succinyl inhibitor (EPO) in PDB entry 1CSB [6]. **a** shows the partitioning of EPO into structural elements. Chains are boxed in green, rings in blue, isolated atoms in red. The simple orthographic projection of the 3D structure of EPO is shown in **b**. **c** presents the ligand layout after the first step of the ligand layout algorithm. A conflict occurs with several atom overlaps and bond crossings. **d** shows the ligand layout (in black) obtained after the conflict resolution step and the processing of the explicit residue (Cystein 29 - chain A). The explicit residue appears in grey and the connection in red. **e** is the diagram obtained after the interaction layout algorithm. Hydrogen bonds are in blue and nearby residues are represented by ovals coloured depending on hydrophobic properties (polar residues in pink, non-polar in green and other in grey). For each residue the chain ID encoding in the PDB file is given in brackets. **f** is the final diagram produced by LeView, containing the residue names.

In LeView, ligands are encoded by undirected labelled graphs in which nodes represent atoms, edges correspond to chemical bonds and labels are atom names. A graph mapping algorithm is used to map each ligand residue with the corresponding PDBeChem cif file to obtain the bond order as PDB files do not contain this information. The ligand layout algorithm is then applied to obtain the 2D coordinates. First, the ligand is partitioned into structural elements: rings (the smallest set of smallest rings), chains containing at least 3 connected non-terminal atoms (*i.e.* C, N, O, S), isolated atoms and terminal atoms. Figure 2a shows the partitioning of epoxy succinyl inhibitor (EPO) of PDB entry 1CSB [6]. Once the ligand has been partitioned a set of rules are applied to each structural element in order to obtain an aesthetic diagram, *i.e.* bond lengths and angles following chemical conventions. For example, angles located in chains are set to 120 degrees and in rings angles and bond lengths are fixed to obtain regular polygons with internal angle equal to 360 degrees divided by the number of atoms in the ring. When none of atoms including in the processed structural element is fixed in a previous step, the initial orientation is kept, for example, atom groups bound to a ring.

For each structural element, all the atoms and their neighbours are recursively placed. The recursive algorithm starts with the longest chain or with the larger ring otherwise. In the case of EPO (Figure 2a), algorithm starts with CG1 atom, one of the terminal atom of the longest chain, keeping the initial orientation of the chain. CB, one of the CG1 neighbours, is then processed followed by its neighbours (CG2 and CA). All the chain atoms are recursively processed with their neighbours (C, N, C4, O4, C3, C2, O2, C1, O1, O5, C5, C6 and CG1). The following atom in the call stack is C: 2D coordinates of C atom are previously calculated and are then calculated for its neighbours O and N2 atoms. when N2 atom, which is included in a ring, is processed the next step place all the ring atoms (CD3, CG3, CB1, and CA1). C7 atom is finally placed with their neighbours OXT and O3. Figure 2b shows the simple orthographic projection of the 3D structure of EPO and Figure 2c shows the resulting EPO coordinates obtained with the ligand layout algorithm.

Keeping the initial orientation of structural elements could lead to atom clashes and bond crossings. For example, Figure 2c shows that some conflicts occur. The next step of the ligand layout algorithm tries to resolves conflicts. A conflict is detected when the distance between two atoms is less than 0.4 Å. In this case, all torsion angles which do not imply ring atoms are flipped (0 to 180 degrees) if this change reduces the overlaps in the molecule. An explicit residue, the cystein 29 of chain A (CYS29) is connected to the ligand in the PDB entry 1CSB through a LINK record wich specify connectivity between residues that is not implied by the primary structure. The ligand layout algorithm is applied to the explicit residue CYS29. Once the residue 2D coordinates are obtained, the residue is randomly placed on a circle with a radius of the real 3D distance. The number of bond crossings and atoms overlaps with the ligand are calculated for each position obtained by rotating the residue through 10 degrees each time. The returned position is the position minimising the number of bond crossings and atom overlaps. Figure 2d shows the resulting conflict-free layout of EPO and the explicit residue CYS29.

**Figure 3:**
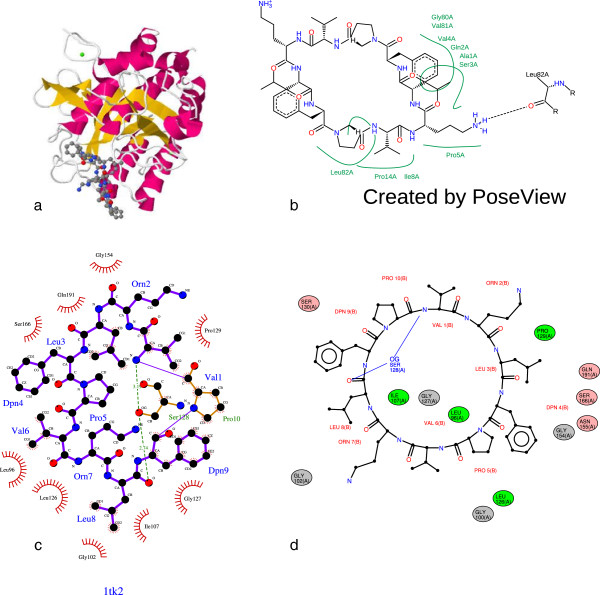
**Diagrams produced by LigPlot+, PoseView and LeView for Gramicidin S (PDB entry 1TK2). a** shows the 3D coordinates at the interface ligand/protein for PDB entry 1TK2. The corresponding diagram produced by the website version of PoseView is shown in **b**, by LigPlot+ using default settings in **c** and by LeView using default settings in **d**.

During LeView implementation, a special attention has been paid to macro-cycles, *i.e.* large rings with smaller rings fused or bridged to them. Macro-cycles are detected and atoms forming the macro-cycle are first processed in order to form a regular polygon to produce a clear diagram. Finally the smaller rings and the others structural elements are processed. Figure 3d shows the diagram produced by LeView for the gramicidin S (1TK2 PDB entry) containing a macro-cycle.

### Interaction layout

A list of neighbouring residues that have at least one non-hydrogen atom within a cut-off distance of 4 Å by default from any ligand atom is constructed. Hydrogen-bond donor and acceptor atoms in residues on this list that lie within a cut-off distance to a ligand atom are identified. Donor and acceptor atoms are also identified in the ligand. For each pair of donor(D)/acceptor (A) atoms, a hydrogen bond is created if an angle (D,A,aa) is less than 90 degrees exists (with aa, an acceptor’s neighbour atom). For water-mediated hydrogen bonds, water molecules within 3.3 Å of the donor/acceptor atoms in the ligand are identified as well as all paths between a ligand atom and a residue atom containing between one and four water molecules. Neighbouring residues are defined as biomacromolecule residues which have at least one heavy atom approaching within a cut-off distance to the ligand. Once all interactions are identified, the next step is to place all interactions around the ligand, minimising bond crossings and overlapping graphical elements. The same method is used for placing all interaction types. The interacting element is randomly placed on a circle with a radius of the real 3D distance. The number of bond crossings and nearby atom, *i.e.* atoms approaching within a distance less than 0.4 Å are calculated for each position obtained by rotating the interacting element through 5 degrees steps. The best position is that which minimises the number of bond crossings. If several positions show the same minimum number of bond crossings, the selection is made based on the position that has minimal close interactions. LeView first places the hydrogen bonds, then the nearby residues and finally any water-mediated hydrogen bonds. LeView starts to place hydrogen bonds because they are represented by lines implying a higher probability of atom overlaps and bond crossings than nearby residues which are represented by ovals and placed in second. Water-mediated hydrogen bonds are not displayed by default and when the user add one, the coordinates are calculed to include it on the existing diagram. Figure 2e shows the layout of hydrogen bonds and nearby residues in the example of EPO ligand.

### Text layout

The finally step of the layout algorithm is to add text on the diagram. The name of each residue component of the ligand and the name of explicit residues are placed on the diagram in order to minimise overlaps with other diagram elements. The residue name is randomly placed on a circle with the centre being the centroid of the residue which is the arithmetic mean position of all the residue atoms and a radius of *Max*(*distance*(*centroid*,*i*))+0.5 with *i* the residue atoms, *i.e.* the distance between the centroid and the further residue atom plus 0.5 Å. The number of nearby elements approaching within a distance less than 2 Å is calculated for each position obtained by rotating the text through 10 degrees each time. The position minimising the number of nearby elements is kept. Figure 2f shows the final diagram generated with default settings by LeView for EPO in the PDB entry 1CSB.

## Results and discussion

LeView automatically detects biomacromolecules, ligands and metal ions in a PDB file and shows the user a complete list of these. Composite ligands can also be user specified by entering the appropriate residue range. It is possible to create an interactive 2D diagram for each ligand and metal ion and this shows ligands, metals, hydrogen bonds and nearby residues. Nearby residues are defined as biomacromolecule residues (*i.e.* in proteins, DNA or RNA) which have at least one heavy atom approaching within a cut-off distance to the ligand. LeView allows the user to display several ligands and metal ions from the same or from several different PDB entries, which makes it easy to compare diagrams. Figure 2f shows the 2D diagram produced by LeView using default settings for EPO in PDB entry 1CSB. LeView offers the user a highly functional and intuitive interactive graphical interface to customise the 2D diagram. The user can change the cut-off distance for hydrogen bonds and nearby residues by sliding the corresponding distance cut-off bar. Possible water-mediated interactions involving the ligand or ion, with up to four bridging water molecules, are not displayed by default but can be added to the diagram through the menu. A number of display options are available as well. Atom labels (*e.g.* C15) can be displayed or hidden and the user can choose between standard colours (*e.g.* red for oxygen) or plain-colour mode, *i.e.* ligand atoms appear in the same colour than the ligand chemical bonds while several colour schemes are available for nearby residues. Colour can be used to represent hydrophobicity/hydrophilicity, standard amino acid properties similar to the Shapely scheme available in RasMol [7], charge or secondary structure type. The colour of every element in the diagram can be changed via the colour menu. Hydrogen bonds can be represented by solid lines (default) or arrows from donor to acceptor, and hydrogen-bond distances additionally displayed. LeView allows the user to reposition all the graphical elements in the diagram at will. If desired, individual hydrogen bonds and nearby residues can be deleted from the diagram, by a right click on it. Finally, the diagram can be exported in a variety of raster and vector graphics formats: PNG, GIF, JPG, PDF, SVG and EPS. For the latter three formats, LeView uses the VectorGraphics package of the FreeHEP Java Library (http://java.freehep.org). The list of the interactions represented in the diagram, with the atoms involved and the associated distance, can also be exported as a flat text.

**Figure 4:**
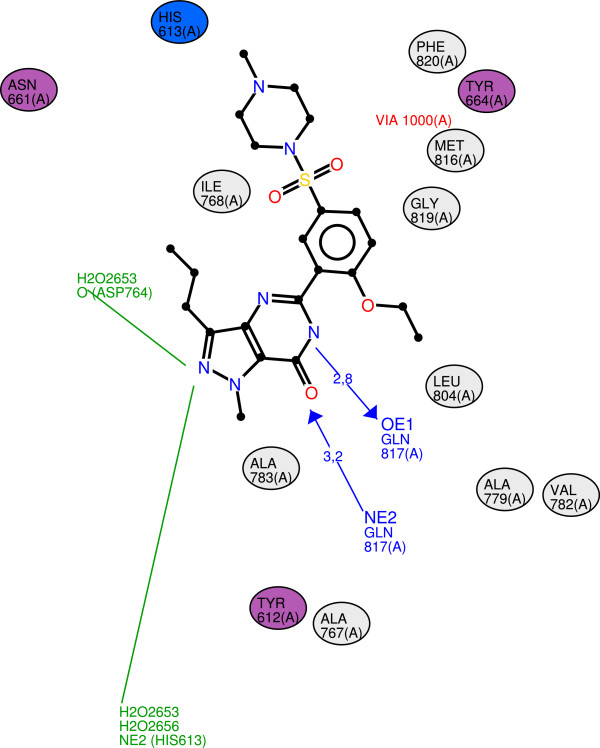
**Example of user-customised diagram produced by LeView.** Figure 4 shows an example of diagram generated by LeView for the PDE5 inhibitor sildenafil (PDB entry 1UDT [8]) after user customisation. The ligand appears in black. Hydrogen bonds are represented by blue arrows from donor to acceptor and the distance is displayed. Nearby residues are represented by ovals coloured depending on charge properties (non-polar residues in grey, acidic residues in red, basic residues in blue and uncharged polar residues in purple). Two water-mediated hydrogen bonds appear in green. The number of water molecules is indicated by the number of H2O.

Table 1 shows the main features of the non-commercial programs to generate 2D diagrams of protein-ligand interactions. These include LigPlot+, the website version of PoseView and LeView. LeView is the only program distributed under GNU General Public Licence and free for both academic and non-academic users. It is also the only tool with a wide range of output formats. LigPlot+ and LeView are able to automatically detect ligands from the PDB file whereas PoseView need a distinct ligand file. Figure 3 shows the diagrams produced by the three programs for gramicidin S (PDB entry 1TK2) which is composed of ten residues forming a macro-cycle. In this example, LigPlot+ does not correctly detect the entire ligand: the proline (residue 10 of chain B) is detected as an explicit residue (Figure 3c) whereas LeView correctly identified all residues make up gramicidin S (Figure 3d). Contrary to other tools, LeView pays a special attention to macro-cycles makes the resulting diagram very clear as shown in Figure 3d. The graphical user interface implemented in LeView makes it very easy to use and allows the user to customise the diagram. Figure 4 is an example of user-customised diagram produced by LeView for the the PDE5 inhibitor sildenafil (PDB entry 1UDT [8]). Hydrogen bonds are represented by arrows and the distance is shown. LeView is the only program that can display water-mediated hydrogen bonds through the graphical interface. Two of them are shown in Figure 4. LeView software uses the Java compile once and run everywhere paradigm and can produce high-quality and clear diagrams which are useful and easily understood by scientist without an expert knowledge of structural biology or chemistry.

**Table 1:** Main features of non-commercial programs to generate 2D diagrams of protein-ligand interactions

	**LigPlot+**	**PoseView**	**LeView**
		**website version**	
reference	[1,2]	[3]	this paper
availability	academic licence	free	GNU licence
	commercial licence		
ligand detection	+	-	+
number of entries	several entries	1	several entries
	and alignment		
GUI	+	-	+
customisation	+	-	+
type of	hydrogen bonds,	hydrogen bonds,	hydrogen bonds,
interactions	hydrophobic interactions	hydrophobic interactions	nearby residues,
			water-mediated H-bonds
output format	PS	PDF	PNG, JPG, GIF, PDF
			SVG, EPS, flat text

This table shows exclusively non-commercial programs. A commercial standalone version of PoseView is available but only the free website version of PoseView is considered here. GUI means Graphical User Interface.

## Conclusion

LeView produces customisable and high-quality figures often necessary for scientific publications, in several popular formats. It is able to work with a wide range of complex ligands and environments not possible with existing tools. This tool can be freely downloaded and easily used without installation and offers the user an interactive graphical interface with numerous options to customise the figure such as: varying the cut-off distances, changing the diagram element colours, moving and deleting elements. The diagrams are a good compromise between a faithful representation of the 3D data (structures and interactions) and aesthetic criteria. Ligand, hydrogen bonds and nearby residues are included and the list of possible water-mediated interactions is also available.

## Availability and requirements

**Project name:** LeView**Project home page:**http://www.pegase-biosciences.com/tools/leview/**Operating system(s):** Platform independent**Programming language:** Java**Other requirements:** Java 1.5 or higher**License:** GNU General Public Licence **Any restrictions to use by non-academics:** no restrictions

## Competing interests

The author declared that she has no competing interest.

## Authors’ contributions

SC has developed and maintains the LeView software.

## Supplementary Material

Additional file 1 **The following additional data are available with the online version of this paper.** Additional data file 1 is an archive of the source code of the current version of LeView. Click here for file
