# Supplementary material for: LeView: automatic and interactive generation of 2D diagrams for biomacromolecule/ligand interactions
Source: J Cheminform. 2013 Aug 29;5:40. doi: 10.1186/1758-2946-5-40 (PMC3765711; doi:10.1186/1758-2946-5-40)
Supplement: Additional file 1 — The following additional data are available with the online version of this paper. Additional data file 1 is an archive of the source code of the current version of LeView. [file 1758-2946-5-40-S1.zip › LeView-src/src/html/delete.html~]

Help


# Deleting interactions

LeView allows the user to delete
hydrogen bonds or close residues.

To delete an interaction, **right click** on the interaction you want to delete and choose the **"delete"** item.
