# Supplementary material for: LeView: automatic and interactive generation of 2D diagrams for biomacromolecule/ligand interactions
Source: J Cheminform. 2013 Aug 29;5:40. doi: 10.1186/1758-2946-5-40 (PMC3765711; doi:10.1186/1758-2946-5-40)
Supplement: Additional file 1 — The following additional data are available with the online version of this paper. Additional data file 1 is an archive of the source code of the current version of LeView. [file 1758-2946-5-40-S1.zip › LeView-src/src/html/cutoff.html~]

Help


# Changing cut-off

LeView allows the user to change the cut-off distance for hydrogen bonds and close residues. The initial cut-off distance for hydrogen bonds is 3.3 A for hydrogen bonds and 4.0 A for close residues. These can be changed by sliding the corresponding cut-off bar at the bottom of the frame.

While you are in the "move elements" mode, the maximum cut-off distance remains fixed at the one chosen before you switched on this mode. You will need to deactivate this mode in order to increase the cut-off distance.
