# Supplementary material for: LeView: automatic and interactive generation of 2D diagrams for biomacromolecule/ligand interactions
Source: J Cheminform. 2013 Aug 29;5:40. doi: 10.1186/1758-2946-5-40 (PMC3765711; doi:10.1186/1758-2946-5-40)
Supplement: Additional file 1 — The following additional data are available with the online version of this paper. Additional data file 1 is an archive of the source code of the current version of LeView. [file 1758-2946-5-40-S1.zip › LeView-src/src/html/waterMediated.html]

Help


# Water mediated hydrogen bonds

LeView allows the user to display possible water-mediated interactions involving the ligand or ion. By clicking on the **"Water Mediated"** menu, all the possible water-mediated hydrogen bonds are displayed (limited to four H2O). Click on one of them to display it on the diagram and click on again to hide it.
