# Supplementary material for: LeView: automatic and interactive generation of 2D diagrams for biomacromolecule/ligand interactions
Source: J Cheminform. 2013 Aug 29;5:40. doi: 10.1186/1758-2946-5-40 (PMC3765711; doi:10.1186/1758-2946-5-40)
Supplement: Additional file 1 — The following additional data are available with the online version of this paper. Additional data file 1 is an archive of the source code of the current version of LeView. [file 1758-2946-5-40-S1.zip › LeView-src/src/html/moving.html~]

Help


# Moving elements

LeView allows the user to move all the diagram elements. To do this, click on the **"Move elements"** button at the top of the frame.

While this button is pressed, you can move each diagram element by clicking on it and releasing it to a new position. In this mode, the cut-off distance for H-bonds and close residues can be decreased, but not increased. In order to increase the cut-off distance, you will need to come out of **"Move elements"** mode by clicking the **"cancel"** button; however, doing this may mean that you lose your position modifications.
