# Supplementary material for: LeView: automatic and interactive generation of 2D diagrams for biomacromolecule/ligand interactions
Source: J Cheminform. 2013 Aug 29;5:40. doi: 10.1186/1758-2946-5-40 (PMC3765711; doi:10.1186/1758-2946-5-40)
Supplement: Additional file 1 — The following additional data are available with the online version of this paper. Additional data file 1 is an archive of the source code of the current version of LeView. [file 1758-2946-5-40-S1.zip › LeView-src/src/html/options.html~]

Help


# Options

LeView offers the user a number of options.

## Atom labels

The user can choose to display or hide the atom labels (e.g. C19).

## Colour scheme for atoms

The user can choose between standard colours (e.g. red for oxygen) or plain-colour mode.

## Colour scheme for close residues

The user can choose between several colour schemes.

- The **Hydro. scheme** represents the residue according to its hydrophobic properties: polar residues in pink, non-polar in green and others in gray
- The **Prop.** scheme represents residues depending on the standard amino acid properties. Bright colours are used for polar residues and darker colours for non-polar ones. This colour scheme is similar to the Shapely scheme that is available under the RasMol Colours menu.
- The **Charge scheme** represents residues depending on the charge. non-polar residues gray, acidic residues red, basic residues blue and uncharged polar residues purple.
- The **Struct. scheme** represents residues depending on the secondary structure in which they are included: Pink for helix, yellow for strand and white for other.

## Element colours

The user can define the colour for each element of the diagram. To do this, go to the **Element Colour** submenu and click on the element for which you want to change the colour. This will open a colour frame.

## Hydrogen bond display

The user can choose between two **hydrogen bond styles**:

- lines: H bonds are represented by simple lines
- arrows: H bonds are represented by arrows from donor to acceptor.

The user can choose to display or hide the hydrogen bond distance.
