# Supplementary material for: LeView: automatic and interactive generation of 2D diagrams for biomacromolecule/ligand interactions
Source: J Cheminform. 2013 Aug 29;5:40. doi: 10.1186/1758-2946-5-40 (PMC3765711; doi:10.1186/1758-2946-5-40)
Supplement: Additional file 1 — The following additional data are available with the online version of this paper. Additional data file 1 is an archive of the source code of the current version of LeView. [file 1758-2946-5-40-S1.zip › LeView-src/src/html/about.html~]

Help


# About the method

## Ligand Layout

In the first step, the ligand is split into structural elements:

- **Rings**: the smallest of smallest rings
- **Chains**: containing at least 3 non-terminal atoms (C, N, O, S)
- **Isolated atoms**
- **Terminal atoms**

Once the ligand has been partitioned, a set of rules is applied for each structural element in order to obtain an aesthetic diagram, i.e. ideal bond lengths and angles. We keep the initial orientation from 3D coordinates for each element. If any conflicts occur, we try to resolve them by rotating the bonds.

## Close Residues

Close residues are residues which have at least one heavy atom approaching within a cut-off distance to the ligand.

## Hydrogen Bonds

The hydrogen bond donor and the hydrogen bond acceptor atoms are identified in the ligand. Then, donor and acceptor atoms in residues approaching within a cut-off distance to the ligand atoms are identified. For each pair of donor (D)/acceptor (A) atoms, a hydrogen bond is created if an angle (D,A,aa) less than 90 degrees exists (with aa, an acceptor's neighbour atom).

## Water-mediated hydrogen bonds

The hydrogen bond donor and the hydrogen bond acceptor atoms are identified in the ligand. Next, water molecules approaching within 3.3Å to the ligand atoms are identified. Then all the paths between a ligand atom and a residue atom containing between 1 and 4 water molecules are calculated.

## Layout of interactions

The same method is used for all interaction types. The element is randomly placed on the circle with a radius of the real distance. A score, depending on the numbers of bond crossings and close atoms, is calculated for each position obtained by rotating the element through 5 degrees each time. The position corresponding to the best score is kept. We begin by placing the hydrogen bonds, then the close residues and finally, the water-mediated hydrogen bonds.
