# Supplementary material for: LeView: automatic and interactive generation of 2D diagrams for biomacromolecule/ligand interactions
Source: J Cheminform. 2013 Aug 29;5:40. doi: 10.1186/1758-2946-5-40 (PMC3765711; doi:10.1186/1758-2946-5-40)
Supplement: Additional file 1 — The following additional data are available with the online version of this paper. Additional data file 1 is an archive of the source code of the current version of LeView. [file 1758-2946-5-40-S1.zip › LeView-src/src/html/index.html]

Help


# LeView

## About this tool

LeView (Ligand Environment Viewer) is a Java program that can be used to generate 2D representations of ligands and their environments and binding interactions for PDB entries. The program can be used automatically or interactively - in the latter case, you can customise the display in a variety of ways. The views you create can be saved in several different graphics formats.

First, it automatically detects biomacromolecules, ligands and metals in the PDB file. Then it generates a list of ligands and metals existing in the PDB entry. For each ligand and metal you can obtain an interactive 2D diagram. This diagram shows ligands, metals, hygrogen bonds, close residues, explicit residues and metals.

The user can easily manipulate the diagram by changing the cut-off distance for hydrogen bonds and close residues. Probable water-mediated hydrogen bonds can be added to the diagrams (see the section "Water mediated H bonds"). The diagram can also be manipulated by moving elements (see the section on "Moving elements") or by choosing the element colour and style (see the section on "Options"). The diagram can be exported in different formats such as PNG, PDF, SVG or EPS (see the section on "Export").
